# Supplementary material for: Sodium and Salt Consumption in Latin America and the Caribbean: A Systematic-Review and Meta-Analysis of Population-Based Studies and Surveys
Source: Nutrients. 2020 Feb 20;12(2):556. doi: 10.3390/nu12020556 (PMC7071427; doi:10.3390/nu12020556)
Supplement: Supplementary file 1 [file nutrients-12-00556-s001.zip › Supplementary Material 20200106 v02 [Nutrients] Proofs.docx]

**Sodium and salt consumption in Latin America and the Caribbean: A systematic-review and meta-analysis of population-based studies and surveys**

# Table S1: PRISMA checklist

| **Section/topic** | **#** | **Checklist item** | **Reported on page #** |
| --- | --- | --- | --- |
| **TITLE** | | |  |
| Title | 1 | Identify the report as a systematic review, meta-analysis, or both. | 01 |
| **ABSTRACT** | | |  |
| Structured summary | 2 | Provide a structured summary including, as applicable: background; objectives; data sources; study eligibility criteria, participants, and interventions; study appraisal and synthesis methods; results; limitations; conclusions and implications of key findings; systematic review registration number. | 03 |
| **INTRODUCTION** | | |  |
| Rationale | 3 | Describe the rationale for the review in the context of what is already known. | 04 |
| Objectives | 4 | Provide an explicit statement of questions being addressed with reference to participants, interventions, comparisons, outcomes, and study design (PICOS). | 04 |
| **METHODS** | | |  |
| Protocol and registration | 5 | Indicate if a review protocol exists, if and where it can be accessed (e.g., Web address), and, if available, provide registration information including registration number. | 05 |
| Eligibility criteria | 6 | Specify study characteristics (e.g., PICOS, length of follow-up) and report characteristics (e.g., years considered, language, publication status) used as criteria for eligibility, giving rationale. | 05 |
| Information sources | 7 | Describe all information sources (e.g., databases with dates of coverage, contact with study authors to identify additional studies) in the search and date last searched. | 05 |
| Search | 8 | Present full electronic search strategy for at least one database, including any limits used, such that it could be repeated. | 05 |
| Study selection | 9 | State the process for selecting studies (i.e., screening, eligibility, included in systematic review, and, if applicable, included in the meta-analysis). | 06 |
| Data collection process | 10 | Describe method of data extraction from reports (e.g., piloted forms, independently, in duplicate) and any processes for obtaining and confirming data from investigators. | 06 |
| Data items | 11 | List and define all variables for which data were sought (e.g., PICOS, funding sources) and any assumptions and simplifications made. | 06 |
| Risk of bias in individual studies | 12 | Describe methods used for assessing risk of bias of individual studies (including specification of whether this was done at the study or outcome level), and how this information is to be used in any data synthesis. | 06 |
| Summary measures | 13 | State the principal summary measures (e.g., risk ratio, difference in means). | 07 |
| Synthesis of results | 14 | Describe the methods of handling data and combining results of studies, if done, including measures of consistency (e.g., I^2^) for each meta-analysis. | 07 |
| Risk of bias across studies | 15 | Specify any assessment of risk of bias that may affect the cumulative evidence (e.g., publication bias, selective reporting within studies). | NA |
| Additional analyses | 16 | Describe methods of additional analyses (e.g., sensitivity or subgroup analyses, meta-regression), if done, indicating which were pre-specified. | NA |
| **RESULTS** | | |  |
| Study selection | 17 | Give numbers of studies screened, assessed for eligibility, and included in the review, with reasons for exclusions at each stage, ideally with a flow diagram. | 08 |
| Study characteristics | 18 | For each study, present characteristics for which data were extracted (e.g., study size, PICOS, follow-up period) and provide the citations. | 08 |
| Risk of bias within studies | 19 | Present data on risk of bias of each study and, if available, any outcome level assessment (see item 12). | NA |
| Results of individual studies | 20 | For all outcomes considered (benefits or harms), present, for each study: (a) simple summary data for each intervention group (b) effect estimates and confidence intervals, ideally with a forest plot. | 09 |
| Synthesis of results | 21 | Present results of each meta-analysis done, including confidence intervals and measures of consistency. | 08-09 |
| Risk of bias across studies | 22 | Present results of any assessment of risk of bias across studies (see Item 15). | NA |
| Additional analysis | 23 | Give results of additional analyses, if done (e.g., sensitivity or subgroup analyses, meta-regression [see Item 16]). | NA |
| **DISCUSSION** | | |  |
| Summary of evidence | 24 | Summarize the main findings including the strength of evidence for each main outcome; consider their relevance to key groups (e.g., healthcare providers, users, and policy makers). | 10 |
| Limitations | 25 | Discuss limitations at study and outcome level (e.g., risk of bias), and at review-level (e.g., incomplete retrieval of identified research, reporting bias). | 11-12 |
| Conclusions | 26 | Provide a general interpretation of the results in the context of other evidence, and implications for future research. | 13 |
| **FUNDING** | | |  |
| Funding | 27 | Describe sources of funding for the systematic review and other support (e.g., supply of data); role of funders for the systematic review. | 01 |

*From:*  Moher D, Liberati A, Tetzlaff J, Altman DG, The PRISMA Group (2009). Preferred Reporting Items for Systematic Reviews and Meta-Analyses: The PRISMA Statement. PLoS Med 6(7): e1000097. doi:10.1371/journal.pmed1000097

# Search terms

## Search terms in Ovid (Medline, Embase and Global Health)

| 1 | exp animals/ not humans.sh. |
| --- | --- |
|  |  |
| 2 | salt.mp. |
| 3 | NaCl.mp. |
| 4 | sodium.mp. |
| 5 | exp Sodium Chloride, Dietary/ |
| 6 | exp Sodium, Dietary/ |
| 7 | 2 … or … 6 |
| 8 | intake.mp. |
| 9 | ingest*.mp. |
| 10 | consum*.mp. |
| 11 | diet*.mp. |
| 12 | urin*.mp. |
| 13 | excret*.mp. |
| 14 | eat*.mp. |
| 15 | 8 … or … 14 |
| 16 | 7 and 15 |
| 17 | (("Antigua and Barbuda") or ("Argentina") or ("Bahamas") or ("Barbados") or ("Belize") or ("Bolivia") or ("Brazil") or ("United States Virgin Islands") or ("British Virgin Islands") or ("Chile") or ("Colombia") or ("Costa Rica") or ("Cuba") or ("Dominica") or ("Dominican Republic") or ("Ecuador") or ("El Salvador") or ("Grenada") or ("Guatemala") or ("Guyana") or ("Haiti") or ("Honduras") or ("Jamaica") or ("Mexico") or ("Nicaragua") or ("Panama") or ("Paraguay") or ("Peru") or ("Puerto Rico") or ("Saint Kitts and Nevis") or ("Saint Lucia") or ("Saint Vincent and the Grenadines") or ("Suriname") or ("Trinidad and Tobago") or ("West Indies") or ("Uruguay") or ("Venezuela") or ("Latin America") or latin amer$ or ("South America") or south amer$ or ("Central America") or central amer$ or ("Caribbean Region")) |
| 18 | 16 and 17 |
| 19 | 18 not 1 |
| 20 | remove duplicates from 19 |

## Search terms in Scopus

(TITLE-ABS-KEY(salt) OR TITLE-ABS-KEY(NaCl) OR TITLE-ABS-KEY(sodium)) AND (TITLE-ABS-KEY(intake) OR TITLE-ABS-KEY(ingest$) OR TITLE-ABS-KEY(consum$) OR TITLE-ABS-KEY(diet$) OR TITLE-ABS-KEY(urin$) OR TITLE-ABS-KEY(excret$) OR TITLE-ABS-KEY(eat$)) AND (TITLE-ABS-KEY("Antigua and Barbuda") or TITLE-ABS-KEY("Argentina") or TITLE-ABS-KEY("Bahamas") or TITLE-ABS-KEY("Barbados") or TITLE-ABS-KEY("Belize") or TITLE-ABS-KEY("Bolivia") or TITLE-ABS-KEY("Brazil") or TITLE-ABS-KEY("United States Virgin Islands") or TITLE-ABS-KEY("British Virgin Islands") or TITLE-ABS-KEY("Chile") or TITLE-ABS-KEY("Colombia") or TITLE-ABS-KEY("Costa Rica") or TITLE-ABS-KEY("Cuba") or TITLE-ABS-KEY("Dominica") or TITLE-ABS-KEY("Dominican Republic") or TITLE-ABS-KEY("Ecuador") or TITLE-ABS-KEY("El Salvador") or TITLE-ABS-KEY("Grenada") or TITLE-ABS-KEY("Guatemala") or TITLE-ABS-KEY("Guyana") or TITLE-ABS-KEY("Haiti") or TITLE-ABS-KEY("Honduras") or TITLE-ABS-KEY("Jamaica") or TITLE-ABS-KEY("Mexico") or TITLE-ABS-KEY("Nicaragua") or TITLE-ABS-KEY("Panama") or TITLE-ABS-KEY("Paraguay") or TITLE-ABS-KEY("Peru") or TITLE-ABS-KEY("Puerto Rico") or TITLE-ABS-KEY("Saint Kitts and Nevis") or TITLE-ABS-KEY("Saint Lucia") or TITLE-ABS-KEY("Saint Vincent and the Grenadines") or TITLE-ABS-KEY("Suriname") or TITLE-ABS-KEY("Trinidad and Tobago") or TITLE-ABS-KEY("West Indies") or TITLE-ABS-KEY("Uruguay") or TITLE-ABS-KEY("Venezuela") or TITLE-ABS-KEY("Latin America") or TITLE-ABS-KEY(latin amer$) or TITLE-ABS-KEY("South America") or TITLE-ABS-KEY(south amer$) or TITLE-ABS-KEY("Central America") or TITLE-ABS-KEY(central amer$) or TITLE-ABS-KEY("Caribbean Region")) AND NOT DBCOLL(medl)

## Search terms in LILACS

((sal) OR (cloruro de sodio) OR (NaCl) OR (sodio)) AND ((consumo) OR (ingesta) OR (dieta) OR (excrecion) OR (comer))

AND

(("Antigua y Barbuda") OR ("Argentina") OR ("Aruba") OR ("Bahamas") OR ("Barbados") OR ("Belice") OR ("Bolivia") OR ("Brasil") OR ("Islas Vírgenes de los Estados Unidos") OR ("Islas Vírgenes Británicas") OR ("Islas Caimán") OR ("Chile") OR ("Colombia") OR ("Costa Rica") OR ("Cuba") OR ("Curazao") OR ("Dominica") OR ("Republica Dominicana") OR ("Ecuador") OR ("El Salvador") OR ("Granada") OR ("Guatemala") OR ("Guyana") OR ("Haití") OR ("Honduras") OR ("Jamaica") OR ("México") OR ("Nicaragua") OR ("Panamá") OR ("Paraguay") OR ("Perú") OR ("Puerto Rico") OR ("San Cristóbal y Nieves ") OR ("Santa Lucía") OR ("San Vicente y las Granadinas ") OR ("Surinam") OR ("Trinidad y Tobago") OR ("Turcas y Caicos ") OR ("Uruguay") OR ("Venezuela") OR ("América Latina") OR ("Latinoamérica") OR ("América del Sur") OR ("Sudamérica") OR ("Suramérica​") OR ("América Central") OR ("Centroamérica") OR ("América del Centro") OR ("Caribe"))

# Table S2: Data collation form

| Author | Publication year | Data year | Country | ISO3 | Type | Sample size | Mean age (years) | Age standard deviation | Men proportion (%) | Mean blood pressure | Hypertension proportion (%) |
| --- | --- | --- | --- | --- | --- | --- | --- | --- | --- | --- | --- |
| Carbajal | 2001 | NA | Argentina | ARG | Community | 1225 |  |  | 37.06 |  | 35.83 |
| Lamelas | 2016 | NA | Argentina | ARG | Community | 6529 | 51.10 | 9.90 | 39.00 |  | 51.60 |
| Lamelas | 2016 | NA | Brazil | BRA | Community | 5323 | 52.20 | 9.40 | 45.20 |  | 52.50 |
| Lamelas | 2016 | NA | Chile | CHL | Community | 668 | 52.00 | 9.40 | 33.70 |  | 41.30 |
| Lamelas | 2016 | NA | Colombia | COL | Community | 4513 | 50.80 | 9.80 | 37.10 |  | 37.70 |
| Moliterno | 2018 | 2012 | Uruguay | URY | Community | 149 | 52.69 | 16.52 | 40.30 |  | 36.23 |
| Del Pozo | 1990 | NA | Ecuador | ECU | Community | 332 |  |  | 47.59 | ~110/75 |  |
| Perin | 2018 | 2016 | Brazil | BRA | Community | 517 | 53.50 | 14.10 | 41.60 |  | 44.50 |
| Mill | 2019 | 2013 | Brazil | BRA | National | 8083 |  |  |  |  |  |
| Lopez-Rodrigez | 2009 | NA | Chile | CHL | Community | 48 | 39.00 | 7.00 |  | 121.5/79.5 |  |
| Bisi | 2003 | 2000 | Brazil | BRA | Community | 1663 | 44.97 | 10.58 | 45.90 |  | 42.69 |
| Cipullo | 2010 | 2005 | Brazil | BRA | Community | 1717 | 55.00 | 14.70 | 48.80 |  | 44.38 |
| Rodrigues | 2015 | NA | Brazil | BRA | Community | 272 | 44.00 | 14.00 | 47.43 |  | 31.25 |
| Costa | 1990 | 1974 | Brazil | BRA | Community | 4565 |  |  |  |  | 11.75 |
| Petermann-Rocha | 2019 | 2010 | Chile | CHL | National | 2913 | 46.47 | 18.54 | 41.74 | 128.4/76.5 |  |
| Carrillo-Larco | 2018 | 2015 | Peru | PER | Community | 409 | 45.70 | 16.20 | 44.00 |  | 17.00 |
| Harris | 2018 | 2013 | Barbados | BRB | National | 364 |  |  | 44.23 |  | 34.10 |
| Campino | 2016 | 2013 | Chile | CHL | Community | 135 | 41.2 | 12 | 48.15 |  |  |

| Author | Publication year | Sodium assessment | Collection time | Sodium (g/day) | Sodium standard deviation | Salt (x2.54) |
| --- | --- | --- | --- | --- | --- | --- |
| Carbajal | 2001 | Results were multiplied by 3 (i.e. 24hrs) | Overnight (from 23hrs to 7hrs) | 3.02 | 0.40 | 7.68 |
| Lamelas | 2016 | Kawasaki formula was used to estimate 24-hour sodium excretion, surrogate of daily sodium consumption | Morning fasting midstream urine sample | 4.66 | 1.35 | 11.84 |
| Lamelas | 2016 | Kawasaki formula was used to estimate 24-hour sodium excretion, surrogate of daily sodium consumption | Morning fasting midstream urine sample | 4.57 | 1.47 | 11.61 |
| Lamelas | 2016 | Kawasaki formula was used to estimate 24-hour sodium excretion, surrogate of daily sodium consumption | Morning fasting midstream urine sample | 4.88 | 1.45 | 12.40 |
| Lamelas | 2016 | Kawasaki formula was used to estimate 24-hour sodium excretion, surrogate of daily sodium consumption | Morning fasting midstream urine sample | 4.89 | 1.48 | 12.42 |
| Moliterno | 2018 | As per urine samples | Participants were instructed to collect a 24-hour urine sample | 3.52 | 1.32 | 8.93 |
| Del Pozo | 1990 |  |  | 4.63 | 0.56 | 11.75 |
| Perin | 2018 | As per urine samples | Participants were instructed to collect a 24-hour urine sample | 4.13 | 1.77 | 10.50 |
| Mill | 2019 | Tanaka formula was used to estimate the 24-hour sodium excretion | Random sample (as long as the participant had gone at least 2 hour without urinating | 3.68 | 1.80 | 9.34 |
| Lopez-Rodrigez | 2009 | Tanaka formula was used to estimate the 24-hour sodium excretion | Random sample | 4.10 | 1.00 | 10.41 |
| Bisi | 2003 | As per urine samples (here multiplied by 2) | 12-hour urine collection | 4.55 | 2.67 | 11.57 |
| Cipullo | 2010 | As per urine samples | 12-hour urine collection | Urinary sodium in normotensive: <100 mEq/l=55.1%; 100-149 mEq/l=25.8%; ≥150 mEq/l=19.1%. Urinary sodium in hypertensive: <100 mEq/l=43.5%; 100-149 mEq/l=29.8%; ≥150 mEq/l=26.7%. | | |
| Rodrigues | 2015 | As per urine samples | Participants were instructed to collect a 24-hour urine sample | 4.06 | 1.64 | 10.31 |
| Costa | 1990 | NaCl was estimated by the antilogarithm of the average log(Na/creatinine) of spot urine samples times 1..60 (adjustment for daily creatinine) times 0.058 | Random sample | 5.09 | 0.25 | 12.93 |
| Petermann-Rocha | 2019 | Tanaka formula was used to estimate the 24-hour sodium excretion | Random sample | 3.88 | 1.20 | 9.86 |
| Carrillo-Larco | 2018 | As per urine samples | Participants were instructed to collect a 24-hour urine sample | 4.40 | 2.10 | 11.18 |
| Harris | 2018 | As per urine samples | Participants were instructed to collect a 24-hour urine sample | 2.66 | 1.64 | 6.76 |
| Campino | 2016 | As per urine samples | Participants were instructed to collect a 24-hour urine sample | 4.16 | 1.65 | 10.57 |

# Table S3: Meta-analysis results including all studies

| Overall pooled mean estimated 24-hour sodium consumption | 4.08 g/day (95% confidence interval: 3.65-4.51, I^2^: 99.9%). Equivalent to 10.36 g/day of salt. |
| --- | --- |
| In national samples | 3.43 g/day (95% confidence interval: 3.12-3.75, I^2^: 99.0%). Equivalent to 8.71 g/day of salt. |
| In community samples | 4.25 g/day (95% confidence interval: 3.66-4.83, I^2^: 99.9%). Equivalent to 10.67 g/day of salt. |
| 24-hour urine sample | 3.82 g/day (95% confidence interval: 3.27-4.37, I^2^: 97.9%). Equivalent to 9.70 g/day of salt. |
| Not 24-hour urine sample | 4.25 g/day (95% confidence interval: 3.68-4.81, I^2^: 99.9%). Equivalent to 10.80 g/day of salt. |
| 24-hour urine sample | 3.82 g/day (95% confidence interval: 3.27-4.37, I^2^: 97.9%). Equivalent to 9.70 g/day of salt. |
| Kawasaki formula only | 3.85 g/day (95% confidence interval: 3.67-4.03, I^2^: 96.4%). Equivalent to 9.78 g/day of salt. |
| Tanaka formula only | 4.75 g/day (95% confidence interval: 4.58-4.91, I^2^: 97.9%). Equivalent to 12.07 g/day of salt. |

# Table S4: Risk of bias assessment

| Author | Publication year | Selection | | | | Comparability | Outcome | | |
| --- | --- | --- | --- | --- | --- | --- | --- | --- | --- |
|  |  | Representativeness of the exposed cohort | Selection of the non-exposed cohort | Ascertainment of exposure | Demonstration that outcome of interest was not present at start of study | Comparability of cohorts on the basis of the design or analysis | Assessment of outcome | Was follow-up long enough for outcomes to occur | Adequacy of follow up of cohorts |
| Carbajal | 2001 | a | a | a | n/a | n/a | a | n/a | n/a |
| Lamelas | 2016 | a | a | a | n/a | n/a | a | n/a | n/a |
| Lamelas | 2016 | a | a | a | n/a | n/a | a | n/a | n/a |
| Lamelas | 2016 | a | a | a | n/a | n/a | a | n/a | n/a |
| Lamelas | 2016 | a | a | a | n/a | n/a | a | n/a | n/a |
| Moliterno | 2018 | a | a | a | n/a | n/a | a | n/a | n/a |
| Del Pozo | 1990 | a | a | a | n/a | n/a | a | n/a | n/a |
| Perin | 2018 | a | a | a | n/a | n/a | a | n/a | n/a |
| Mill | 2019 | a | a | a | n/a | n/a | a | n/a | n/a |
| Lopez-Rodrigez | 2009 | a | a | a | n/a | n/a | a | n/a | n/a |
| Bisi | 2003 | a | a | a | n/a | n/a | a | n/a | n/a |
| Cipullo | 2010 | a | a | a | n/a | n/a | a | n/a | n/a |
| Rodrigues | 2015 | a | a | a | n/a | n/a | a | n/a | n/a |
| Costa | 1990 | a | a | a | n/a | n/a | a | n/a | n/a |
| Petermann-Rocha | 2019 | a | a | a | n/a | n/a | a | n/a | n/a |
| Carrillo-Larco | 2018 | a | a | a | n/a | n/a | a | n/a | n/a |
| Harris | 2018 | a | a | a | n/a | n/a | a | n/a | n/a |
| Campino | 2016 | a | a | a | n/a | n/a | a | n/a | n/a |

NEWCASTLE - OTTAWA QUALITY ASSESSMENT SCALE (<http://www.ohri.ca/programs/clinical_epidemiology/nosgen.pdf>)
